# Traditional versus intensive blood glucose control: long-term target range duration and cardiovascular disease risk and all-cause mortality - a real-world cohort study

**DOI:** 10.3389/fendo.2024.1449925

**Published:** 2024-12-10

**Authors:** Jingdi Zhang, Liuxin Li, Donglei Luo, Zhenyu Huo, Xiaoxue Zhang, Yiran Xu, Jingyuan Jiang, Tiantian Liu, Shouling Wu, Zhe Huang

**Affiliations:** ^1^ School of Public Health, North China University of Science and Technology, Tangshan, China; ^2^ Department of Cardiology, Kailuan General Hospital, Tangshan, China; ^3^ Graduate School, North China University of Science and Technology, Tangshan, China; ^4^ Department of Cardiology, Chengde Central Hospital/Second Clinical College of Chengde Medical University, Chengde, Hebei, China

**Keywords:** fasting blood glucose, time in range, diabetes, cardiovascular outcomes, legacy effect

## Abstract

**Objective:**

For a long time, the dispute about whether improved glycemic control can bring significant benefits has remained unresolved. We aimed to investigate the association of time spent in different fasting glucose target ranges with cardiovascular risk and all-cause mortality in a population with type 2 diabetes (T2DM).

**Method:**

A total of 3460 T2DM patients in the Kailuan cohort were included in this study. The Time in Target Range (TITRE) was calculated as the proportion of time that fasting blood glucose (FBG) was within the usual glycemic control range and the intensive glycemic control range between 2006 and 2016. The Cox proportional hazards regression model analyzed the relationship between TITRE, defined by different glucose ranges, and cardiovascular disease and all-cause mortality.

**Results:**

During a median follow-up of 4.23 years, a total of 360 CVDs and 238 deaths were recorded. After correcting for traditional risk factors, we observed that in the conventional blood glucose control group, each increase of 1 standard deviation in TITRE was associated with a 23% (HR, 0.77; 95%CI, 0.68-0.87) reduction in CVD risk and a 20% reduction in all-cause mortality (HR, 0.80; 95%CI, 0.69-0.92). Similar results were also observed in the intensified blood glucose control group. In the conventional blood glucose control group, participants with TITRE of 50% or more had an absolute incidence rate of CVD of 16.77%, whereas in the intensified blood glucose control group, participants with TITRE of 50% or more had an absolute incidence rate of CVD of 11.82%.

**Conclusions:**

In patients with type 2 diabetes, achieving near-normal blood glucose levels appears to significantly reduce the risk of diabetes-related cardiovascular outcomes.

## Introduction

Elevated fasting blood glucose (FBG) levels increase the risk of cardiovascular diseases (CVD) (1), significantly contributing to the global burden of CVD and mortality. Intensive glycemic control has been a primary strategy in managing type 2 diabetes mellitus (T2DM), aiming to reduce the risk of diabetes-related complications by strictly regulating blood glucose levels. However, the controversy over the significant benefits of intensive glycemic control remains unresolved (2).

Early clinical studies indicated that intensive glycemic control did not significantly reduce the incidence of macrovascular complications in T2DM patients and might even be associated with an increased risk of adverse events (3). The 6-year follow-up results of the ADVANCE study, presented at the 2014 EASD Annual Meeting, further explored the “legacy effect” of intensive glycemic control, showing that this effect diminishes during extended follow-up (4). In contrast, the recent UKPDS 91 study, which included a 10-year follow-up of 3277 surviving participants, confirmed the enduring benefits of intensive glycemic control (5). This phenomenon has prompted a reevaluation of intensive glycemic control strategies.

Traditional management of FBG involves controlling FBG levels within a specified target range at a single time point, without accounting for FBG fluctuations over time. Recently, glucose target time (TITRE) (6, 7), derived from multiple FBG measurements, has emerged as a measure associated with the risk of adverse outcomes in diabetes. Therefore, our objective was to assess the relationship between glucose target time and adverse cardiovascular outcomes and all-cause mortality among individuals with varying degrees of glycemic control.

## Methods

### Study population

Since 2006, all employees and retirees of Kailuan Group have undergone biennial physical examinations conducted by Kailuan General Hospital and its affiliated medical facilities. To date, seven rounds of comprehensive health assessments have been completed.

**Figure 1:**
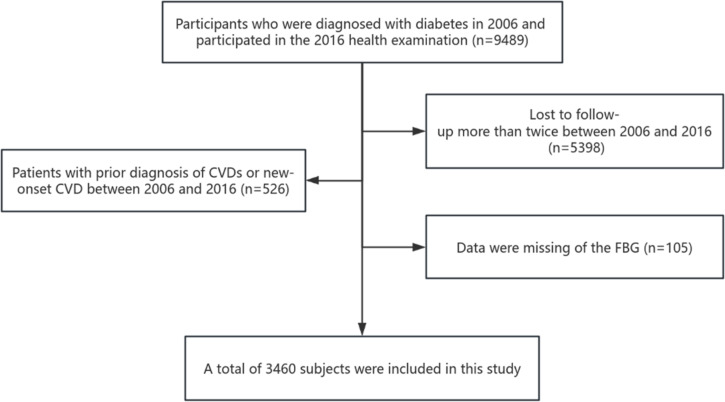
Flow chart of inclusion and exclusion.

This study included 9489 subjects who were diagnosed with diabetes or had a history of diabetes in 2006 and actively participated in health examinations from 2006 to 2016. The time difference between health check-ups in 2006 and 2016 is considered the window period. Subjects were subsequently excluded from the analysis if any of the following criteria applied: (1) Lost to follow-up more than twice between 2006 and 2016 (n=5398); (2) Patients with prior diagnosis of CVDs or new-onset CVD between 2006 and 2016 (n=526); (3) Data were missing of the FBG (n=105). Therefore, 3,460 subjects were deemed eligible for the analysis. The detailed process is shown in **Figure 1**.

This study adheres to the principles of the Helsinki Declaration and has received approval from the Ethics Committee of Kailuan General Hospital (Approval Number: 2006-05). All participants agreed to take part in the study and provided informed written consent.

### Fasting glucose measurement and definition of TITRE

FBG was measured using the hexokinase/glucose-6-phosphate dehydrogenase method (Mind Bioengineering Co Ltd, Shanghai, China) with an upper detection limit of 30.07 mmol/L (8). Five milliliters of fasting cubital venous blood were collected from each participant at 7:00-9:00 a.m. on the same day. Fasting blood glucose was measured by a Hitachi 7600 automatic biochemical analyzer within 4 hours. According to the guideline, the traditional control range of FBG was defined as 4.4mmol/L<FBG<7.0mmol/L (9). The intensive FBG control range was 3.9mmol/L<FBG<6.1mmol/L. The TITRE was calculated by the ratio of the time that the FBG was in the target range during the window period to the total time in the window period. cv-FBG was calculated using FBG data from the window period. FBG variability was calculated using the coefficient of variation as follows: $CV=\frac{SD}{\bar{X}}\times 100\%$ SD is the standard deviation of the FBG values of the subjects and $\bar{x}$ is their mean-FBG.

### Assessment of covariates

Information on age, sex, smoking status, alcohol use status, education, medical history, and medication use was ascertained by standardized questionnaires. Physical examinations, including measurements of height, weight, and blood pressure, were conducted by trained nurses. Body mass index (BMI) was calculated as weight (kg) divided by height squared (m^2^). High-density lipoprotein cholesterol (HDL-C) and Low-density lipoprotein cholesterol (LDL-C) were measured by enzyme colorimetry. Serum creatinine (Scr) was measured using the sarcosine oxidase method, and the estimated glomerular filtration rate (eGFR) was calculated using Scr, which was calculated using the CKD Epidemiology Collaboration (CKD-EPI) equation (10).

Diabetes was defined as FBG ≥ 7.0 mmol/L or history of diabetes or use of antidiabetic drugs (11, 12). Hypertension was defined as systolic blood pressure (SBP) ≥ 140 mmHg and/or diastolic blood pressure (DBP) ≥ 90 mmHg, use of antihypertensive medications, or a self-reported history of hypertension (13).

### Outcome events and follow-up time

The primary outcomes of this study were the first occurrence of cardiovascular disease (CVD) and all-cause mortality. Data on annual CVD events were obtained from the Kailuan Social Security Information System, with trained medical personnel responsible for recording events at designated hospitals. Specialist physicians confirmed all diagnoses based on hospital medical records. The definition of myocardial infarction (MI) followed the criteria from the World Health Organization’s “Multinational Monitoring of Trends and Determinants in Cardiovascular Disease (MONICA)” project, based on clinical symptoms, dynamic changes in cardiac enzyme and biomarker levels, and electrocardiogram (ECG) results [38]. Heart failure (HF) was diagnosed according to the European Society of Cardiology (ESC) guidelines. Stroke was defined based on clinical symptoms and confirmed by computed tomography (CT), magnetic resonance imaging (MRI), or other diagnostic reports [39].

Cardiovascular disease diagnosis data were sourced from the city’s social insurance agency and hospital discharge records, updated annually during the follow-up period. These records covered all participants in the Kailuan study. A specialized team reviewed discharge records from Kailuan General Hospital and its 11 affiliated hospitals each year to identify suspected cardiovascular disease cases. All-cause mortality data were collected from the Provincial Vital Statistics Office and verified by physicians.

The follow-up began in 2016, and it ended upon the occurrence of the first CVD event. In cases where multiple CVD events occurred, the time of the first event was recorded as the endpoint. If no CVD event occurred but death was reported, the follow-up was considered complete at the time of death. For participants who experienced neither a CVD event nor death, the follow-up continued until December 31, 2021.

### Statistical analysis

The observation subjects were divided into 3 groups according to the TITRE (0%, <50%, ≥50%). Normality test for quantitative variables (see **Supplementary Figure A**). Measurement data by normal distribution were expressed as ( $\bar{X}$ ± S), and a one-way analysis of variance was used for comparison among multiple groups. Measurement data not in line with normal distribution were expressed as median (P25, P75), and the Kruskal-Wallis tests were used to compare various groups. Count data were expressed as the number of cases and percentage, and comparison between groups was analyzed using the chi-square test. Standard deviation (SD) and P for trend were used to estimate the trends in the risk of CVDs and all-cause mortality. Restricted cubic spline curves explored the dose-response relationship of TITRE across different glycemic control ranges at the 30th, 60th, and 90th percentiles of the TITRE distribution.

The linear interpolation method was used to estimate TITRE (14). Cox proportional regression models were used to estimate crude hazard ratios (HRs). They adjusted 95% confidence intervals (CIs) for each 1-SD increase in TITRE for the two different target ranges and cardiovascular outcomes, as well as all-cause mortality. The model was adjusted for age, sex, education level, smoking status, drinking status, physical activity, history of hypertension, dyslipidemia, antihypertensive medication, antidiabetic medication, BMI, SBP, and eGFR. The proportional hazards assumption in the Cox model was tested using the Schoenfeld residual test. All scaling assumptions were generally appropriate.

In addition, we investigated the association of each 1-SD increase in TITRE with CVD subtypes separately. HF and MI were combined as a single category for analysis due to the small number of events. In the stratification analysis, multiplicative interaction terms were used to assess the interaction between the randomization arm and the association of TITRE with CVDs and deaths. The random variables included age (>65 years or ≤65 years), sex (male or female), eGFR at baseline (< 90 mL/min/1.73 m^2^ or ≥ 90 mL/min/1.73 m^2^), BMI at baseline (<25kg/m^2^ or ≥25kg/m^2^) and hypertension (yes or no). Finally, we also considered the average blood glucose (mean-FBG) and cv-FBG about TITRE with CVD and all-cause mortality. Pearson correlation analysis assessed the association between these factors and TITRE.

Multiple studies have demonstrated that diabetic patients are often accompanied by elevated levels of CRP, a marker of chronic low-grade inflammation (15, 16), which may serve as an important driver of CVD (17). Therefore, in the sensitivity analysis, generalized estimating equations (GEE) (18) were used to quantify the association between TITRE and repeated measurements of CRP. As CRP does not follow a normal distribution, it was log-transformed for analysis. The purpose of this analysis was to assess the robustness of the primary findings.

All statistical analyses above were performed using SAS 9.4 and Stata 16 software. A two-sided P value<0.05 was considered statistically significant.

## Result

### Baseline characteristics of the subjects

A total of 3,460 participants were enrolled in the analysis, the mean age was 52.39 ± 9.09 years, and 2,726 (78.8%) were men. Among them, 80% of the subjects had participated in five or more health checkups. The basic characteristics after grouping the TITRE according to different ranges of glycemic control are shown in **Tables 1**, **2**. Regardless of the glycemic range, individuals with higher TITRE were younger, had a lower proportion of smokers, were less likely to have hypertension, and had lower levels of BMI and LDL.

**Table 1:** Characteristics according to tertiles of TITRE*.

Characteristics	Total	Tertile 1	Tertile 2	Tertile 3	*P* value
Numbers	3460	1179	1276	1005	
Age, years	52.39 ± 9.09	52.50 ± 8.08	53.33 ± 8.66	51.08 ± 10.49	<0.0001
Male, N (%)	2726 (78.8)	959 (81.3)	955 (74.8)	812 (80.8)	<0.0001
Current smoker, N (%)	1161 (33.6)	421 (35.7)	409 (32.1)	331 (32.9)	0.1412
Current drink, N (%)	1275 (36.8)	461 (39.1)	429 (33.6)	385 (38.3)	0.0100
Salt intake, N (%)	430 (12.4)	154 (13.1)	150 (11.8)	126 (12.5)	0.6137
Education levels, N (%)	602 (17.4)	786 (15.8)	222 (17.4)	194 (19.3)	0.0955
Physical exercise, N (%)	673 (19.5)	229 (19.4)	247 (19.4)	197 (19.6)	0.9889
FBG (mmol/L)	9.02 ± 2.81	10.15 ± 2.85	8.98 ± 2.71	7.75 ± 2.30	<0.0001
BMI (kg/m^2^)	26.26 ± 3.30	26.47 ± 3.24	26.45 ± 3.33	25.77 ± 3.30	<0.0001
eGFR≥90ml/(min·1.73m^2^), N (%)	2274 (65.7)	757 (64.2)	861 (67.5)	656 (65.3)	0.2193
HDL-C (mmol/L)	1.54 ± 0.41	1.53 ± 0.42	1.52 ± 0.41	1.57 ± 0.39	0.0181
LDL-C (mmol/L)	2.46 ± 0.93	2.45 ± 0.95	2.40 ± 0.91	2.54 ± 0.90	0.0022
Hypertension, N (%)	1989 (57.5)	673 (57.1)	748 (58.6)	568 (56.5)	0.5665
Antihypertensive medication, N (%)	640 (18.5)	186 (15.8)	265 (20.8)	189 (18.8)	0.0060
Antidiabetic medication, N (%)	1452 (42.0)	438 (37.2)	559 (43.8)	455 (45.3)	0.0002

BMI, body mass index; eGFR, estimated glomerular filtration rate; FBG, fasting blood glucose; LDL, low-density lipoprotein; HDL, high-density lipoprotein.

*TITRE: TITRE as defined by conventional blood glucose ranges.

**Table 2:** Characteristics according to tertiles of TITRE*.

Characteristics	Total	Tertile 1	Tertile 2	Tertile 3	*P* value
Numbers	3460	1862	1045	553	
Age, years	52.39 ± 9.09	52.81 ± 8.30	53.54 ± 8.77	48.81 ± 11.15	<0.0001
Male, N (%)	2726 (78.8)	1479 (79.4)	776 (74.3)	471 (85.2)	<0.0001
Current smoker, N (%)	1161 (33.6)	652 (35.0)	328 (31.4)	181 (32.7)	0.1253
Current drink, N (%)	1275 (36.8)	711 (38.2)	354 (33.9)	210 (38.0)	0.0579
Salt intake, N (%)	430 (12.4)	248 (13.3)	119 (11.4)	63 (11.4)	0.2296
Education levels, N (%)	602 (17.4)	300 (16.1)	203 (19.4)	99 (17.9)	0.0731
Physical exercise, N (%)	673 (19.5)	380 (20.4)	207 (19.8)	86 (15.6)	0.0380
FBG (mmol/L)	9.02 ± 2.81	9.66 ± 2.75	8.55 ± 2.73	7.80 ± 2.59	<0.0001
BMI (kg/m^2^)	26.26 ± 3.30	26.53 ± 3.25	26.17 ± 3.25	25.53 ± 3.48	<0.0001
eGFR≥90ml/(min·1.73m^2^), N (%)	2274 (65.7)	1211 (65.0)	720 (68.9)	343 (62.0)	0.0148
HDL-C (mmol/L)	1.54 ± 0.41	1.53 ± 0.41	1.54 ± 0.40	1.59 ± 0.39	0.0059
LDL-C (mmol/L)	2.46 ± 0.93	2.45 ± 0.92	2.42 ± 0.93	2.55 ± 0.92	0.0254
Hypertension, N (%)	1989 (57.5)	1068 (57.4)	618 (59.1)	303 (54.8)	0.2438
Antidiabetic medication, N (%)	640 (18.5)	326 (17.5)	233 (22.3)	81 (14.6)	0.0002
Antihypertensive medication, N (%)	1452 (42.0)	736 (39.5)	457 (43.7)	259 (46.8)	0.0036

BMI, body mass index; eGFR, estimated glomerular filtration rate; FBG, fasting blood glucose; LDL, low-density lipoprotein; HDL, high-density lipoprotein.

*TITRE: TITRE as defined by intensive blood glucose ranges.

### Association of TITRE with outcomes

**Figure 2:**
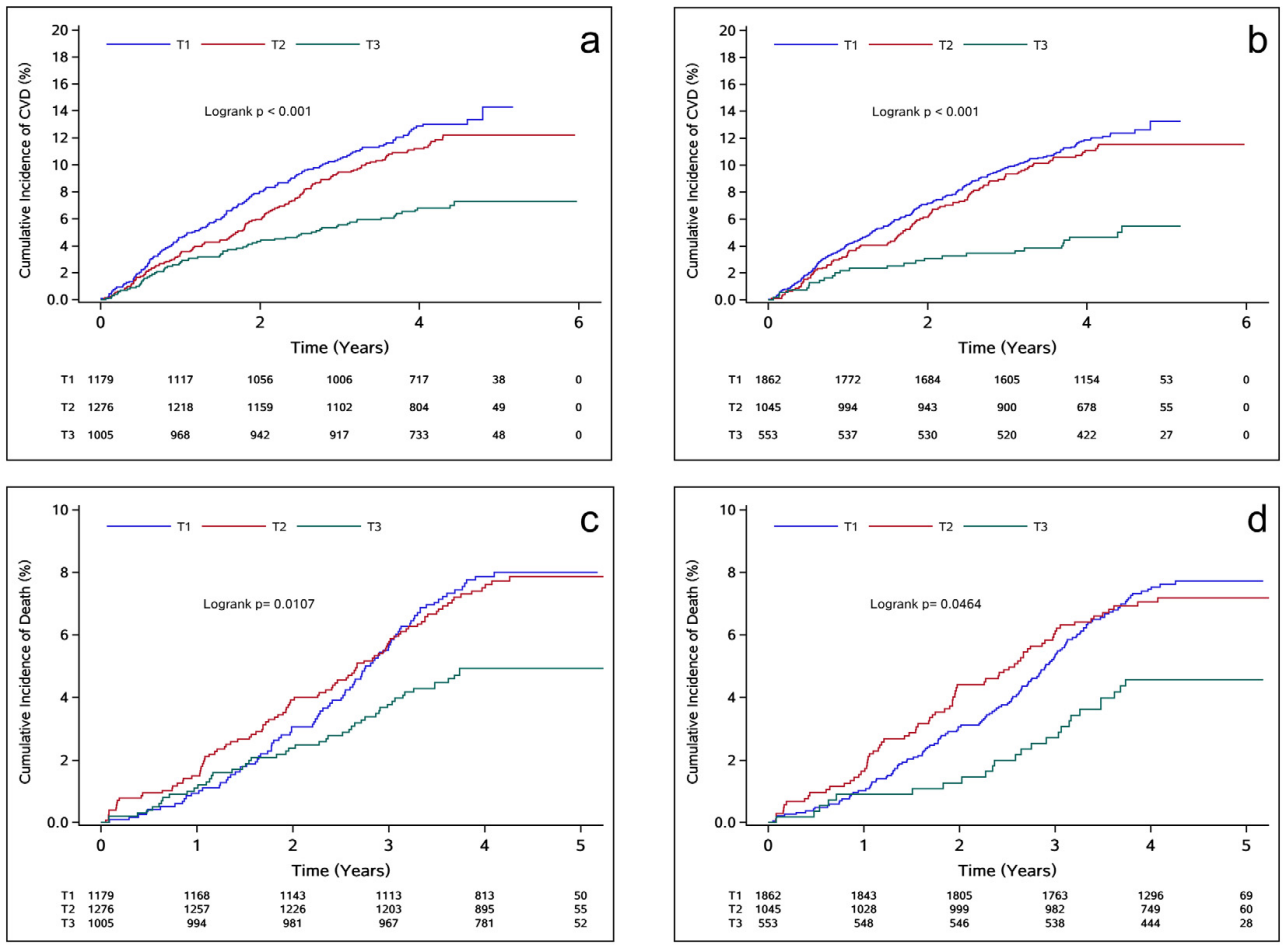
**(A, C)** depict the K-M survival curves focusing on the relationship between TITRE of the traditional group and the observed outcomes; **(B, D)** in order to strengthen group. T1: TITRE=0%; T2: 0%<TITRE<50%; T3: TITRE≥50%.

During a median follow-up of 4.23 years, 360 CVD events and 238 deaths were recorded. Higher TITRE was associated with a decreased cumulative risk of cardiovascular outcomes and all-cause mortality (log-rank test, P < 0.05, **Figure 2**).

**Figure 3:**
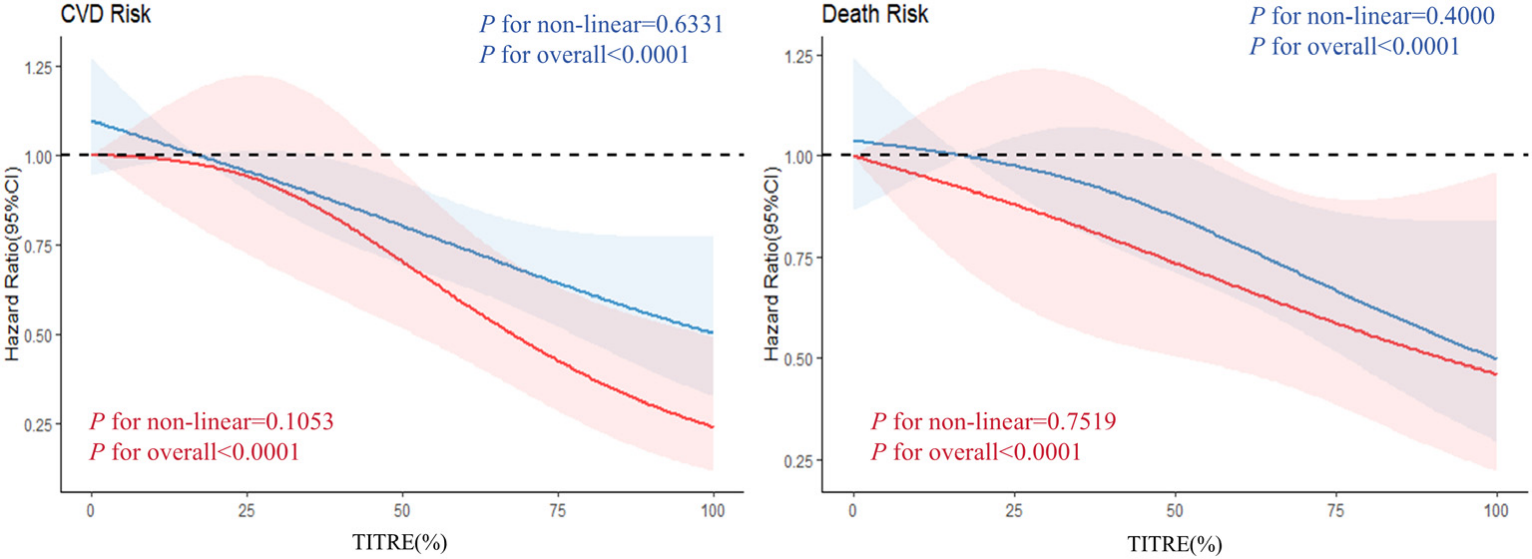
Analyzing the dose-response relationship between different groups of TITRE and the observed outcomes. The red curve represents the intensive group, while the blue curve represents the traditional group.

In the traditional group, the incidence of CVD and all-cause mortality for individuals with TITRE >50% were 16.77 and 11.62 per 1000 person-years, respectively. In the intensified group, the incidence of CVD and all-cause mortality were 11.82 and 10.64 per 1000 person-years, respectively. After adjusting for potential confounding factors, the negative association between TITRE and the risks of CVD and all-cause mortality persisted in both groups. In the traditional group, each 1 SD increase in TITRE was associated with a 23% reduction in the risk of CVD (HR, 0.77; 95% CI, 0.70-0.85; P for trend < 0.0001, **Table 3**) and a 20% reduction in the risk of all-cause mortality (HR, 0.80; 95% CI, 0.73-0.93; P for trend < 0.0001). Similar trends were observed in the intensified group, with an even stronger inverse association noted for cardiovascular disease, as illustrated in **Figure 3**. This association persisted despite further adjustment for cv-FBG in the model. However, it disappeared after correction for mean-FBG. The results of the Pearson correlation test showed a strong negative correlation between the average value of FBG and TITRE (R = -0.76; R = -0.69 in the intensive control group).

**Table 3:** Adjusted hazard ratios for the association of a 1 SD increase in TITRE with cardiovascular outcomes and all-cause mortality.

Outcomes	Event	Model 1		Model 2		Model 3		Model 4	
		HR (95%CI)	*P* value	HR (95%CI)	*P* value	HR (95%CI)	*P* value	HR (95%CI)	*P* value
TITRE as defined by the conventional blood glucose range									
CVD	360	0.76 (0.68, 0.86)	<0.0001	0.77 (0.68, 0.87)	<0.0001	0.77 (0.68, 0.87)	<0.0001	0.91 (0.77, 1.07)	0.2734
All-cause mortality	238	0.80 (0.69, 0.92)	0.0023	0.80 (0.69, 0.92)	0.0023	0.79 (0.69, 0.92)	0.0026	1.09 (0.89, 1.33)	0.3838
TITRE as defined by the intensive blood glucose range									
CVD	360	0.73 (0.64, 0.84)	<0.0001	0.74 (0.65, 0.85)	<0.0001	0.73 (0.63, 0.84)	<0.0001	0.85 (0.72, 1.00)	0.0606
All-cause mortality	238	0.82 (0.70, 0.96)	0.0136	0.82 (0.70, 0.95)	0.0131	0.80 (0.68, 0.94)	0.0075	1.06 (0.87, 1.27)	0.5410

CI, confidence interval; HR, hazard ratio; SD, standard deviation; TITRE, time in target; CVD, cardiovascular disease.

Model 1: Adjust for age and sex.

Model 2: Adjusted for Model1 plus education, smoking status, drinking status, physical activity, history of hypertension, antihypertensive medication, body mass index, systolic blood pressure, High-density lipoprotein cholesterol, Low-density lipoprotein cholesterol, estimated glomerular filtration rate, and antidiabetic medication.

Model 3: Adjusted for Model 2 plus cv-FBG.

Model 4: Adjusted for Model 2 plus mean-FBG.

Consistent results were obtained for CVD subtypes, with a more pronounced negative association between TITRE and stroke in the intensified group (**Table 4**). Each 1 SD increase in TITRE was associated with a 26% reduction in stroke risk in the intensified group, compared to a 20% reduction in the traditional group.

**Table 4:** Hazard ratios associated with CVD subtypes per 1SD increase in TITRE.

Outcomes	Event	Model 1		Model 2		Model 3		Model 4	
		HR (95%CI)	*P* value	HR (95%CI)	*P* value	HR (95%CI)	*P* value	HR (95%CI)	*P* value
TITRE as defined by the conventional blood glucose range									
CVD	97	0.72 (0.57, 0.91)	0.0118	0.73 (0.58, 0.93)	0.0118	0 73 (0.57, 0.92)	0.0104	1.01 (0.73, 1.40)	0.9189
Stroke	188	0.79 (0.68, 0.93)	0.0066	0.80 (0.68, 0.94)	0.0066	0.79 (0.67, 0.94)	0.0069	1.06 (0.84, 1.33)	0.5913
TITRE as defined by the intensive blood glucose range									
CVD	97	0.72 (0.54, 0.95)	0.0219	0.73 (0.55, 0.96)	0.0252	0.72 (0.54, 0.96)	0.0249	0.96 (0.70, 1.32)	0.8310
Stroke	188	0.73 (0.61, 0.89)	0.0016	0.74 (0.61, 0.90)	0.0024	0.72 (0.59, 0.87)	0.0011	0.90 (0.72, 1.13)	0.4025

CI, confidence interval; HR, hazard ratio; SD, standard deviation; TITRE, time in target; CVD, cardiovascular disease.

Model 1: Adjust for age and sex.

Model 2: Adjusted for Model1 plus education, smoking status, drinking status, physical activity, history of hypertension, antihypertensive medication, body mass index, systolic blood pressure, High-density lipoprotein cholesterol, Low-density lipoprotein cholesterol, estimated glomerular filtration rate, and antidiabetic medication.

Model 3: Adjusted for Model 2 plus cv-FBG.

Model 4: Adjusted for Model 2 plus mean-FBG.

The aforementioned model has no multicollinearity among the covariates (VIF < 10).

### Stratification analysis

**Figure 4:**
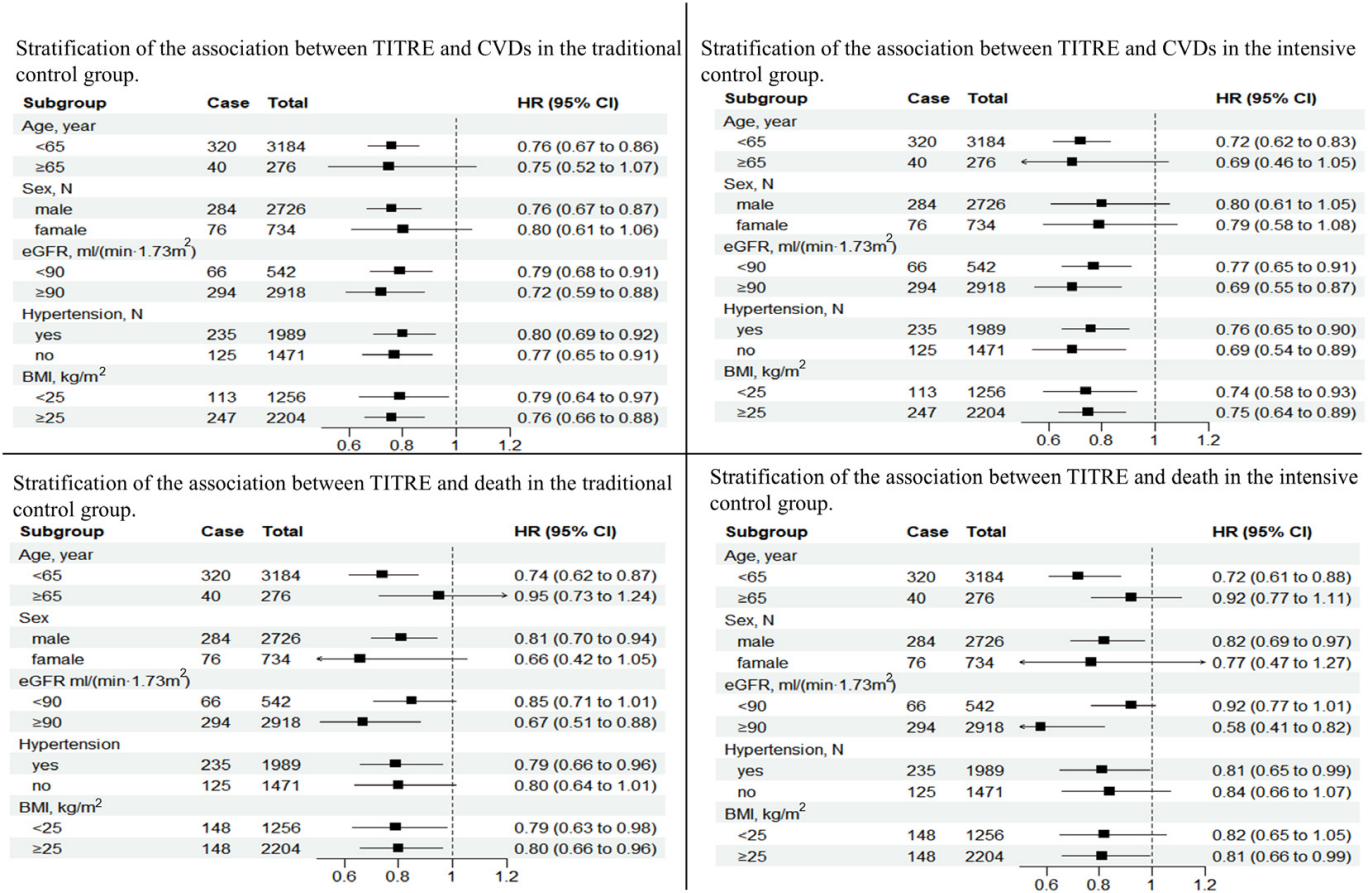
Startified analysis of TITRE and the primary source.

Subgroup analysis showed that the cardiovascular protective effect of TITRE was more significant in male (P for interaction < 0.0001), with each 1-SD increase in TITRE being associated with a 24% reduction in the risk of CVD in the conventional group and a 28% reduction in the enhanced group. Similar results were found in age stratification. In addition, no other significant differences were observed between the groups. However, prolonged intensive glucose control significantly reduced the risk of all-cause death in patients with normal renal function (HR, 0.58; 95% CI, 0.41-0.82, **Figure 4**).

### Sensitivity analysis

In the sensitivity analysis, we applied GEE to assess the relationship between TITRE and repeated measurements of CRP. Since CRP is skewed, we log-transformed CRP in this analysis. The results are shown in **Supplementary Table S1**. In both the conventional and intensive groups, an increase in TITRE was associated with a decrease in log (CRP) values. Notably, this association was more pronounced in the intensive treatment group.

## Discussion

In the Kailuan cohort analysis, after a median follow-up of 4.23 years, we found that long-term control of fasting blood glucose within the intensive target range (TITRE in the intensive group) was associated with a significant reduction in the risk of CVD and all-cause mortality. This inverse association of TITRE with CVD and all-cause mortality was independent of traditional risk factors, including FBG variability.

Our study results indicate a significant linear decreasing trend in the risk of CVD and all-cause mortality with increasing TITRE levels, regardless of the target range. As TITRE levels increase, the risk of CVD and all-cause mortality progressively decreases. Notably, in the intensive glycemic control group, the cardiovascular protective effect in diabetic patients was more pronounced, especially in reducing stroke risk (CVD: HR 0.74, 95% CI 0.65-0.85; stroke: HR 0.74, 95% CI 0.61-0.90). When patients maintained their FBG within the target range for more than 50% of the time, the absolute risk of CVD was reduced by 4.95% in the intensive group compared to the conventional group. The 24-year follow-up of the UKPDS study demonstrated that the legacy effect of early intensive glycemic control did not attenuate during the 24 years after the trial ended (5). The intensive glycemic control group still exhibited significant cardiovascular benefits (a 10% reduction in overall relative risk of death, a 17% reduction in the risk of myocardial infarction, and a 26% reduction in the risk of microvascular complications). This is consistent with our findings. It is important to note that the post-trial follow-up of the UKPDS study lacked detailed glycemic data after the intervention period. In contrast, our study had access to glycemic data. This allowed us to calculate the time within the glycemic target range through a large number of repeated glucose measurements, providing a more comprehensive understanding of long-term glycemic control. Although our study had a shorter follow-up period (median follow-up time of 4.23 years), we preserved detailed glycemic records, allowing for a more in-depth exploration of the sustained impact of glycemic control at different target ranges on cardiovascular outcomes. This fills a critical gap in the UKPDS study results. However, the ADVANCE study, which reported 6-year follow-up results in 2014 (4), showed that intensive glycemic control, compared with standard control, failed to demonstrate long-term benefits in reducing mortality or major macrovascular events. This is inconsistent with our findings, and a possible explanation could be that, after the end of the ADVANCE trial, both groups of patients received similar conventional treatment, leading to a convergence of glucose levels. In contrast, our study grouped patients by their TITRE over ten years, allowing a more intuitive analysis of the relationship between glycemic control and adverse cardiovascular outcomes and all-cause mortality. Additionally, we included heart failure as part of the definition of adverse cardiovascular outcomes, whereas heart failure was not considered in the ADVANCE study. These factors enabled our study to provide a more comprehensive assessment of the potential cardiovascular health benefits of long-term glycemic control.

Moreover, in the stratified analysis of TITRE and the risk of CVD and all-cause mortality, we found that the protective effect of TITRE was more significant in the intensive group, with a larger reduction in the risk of CVD and all-cause mortality. This effect was particularly prominent in the subgroup of patients under 65 years old and those with good renal function. Another interesting finding was that, compared to diabetic patients without hypertension, those with concomitant hypertension who maintained their blood glucose within the target range for a longer period may have a reduced risk of all-cause mortality.

Finally, we also considered the relationship between good glycemic control and the risk of inflammation in the diabetic population. In the sensitivity analysis, repeated measurements of CRP, as an inflammatory marker, were collected to further explore the association between diabetes control and inflammation risk. As glycemic control improved (with increasing TITRE), inflammation levels [log (CRP) values] significantly decreased. This negative correlation was more pronounced under intensive glycemic control (with an estimated coefficient of -0.0025, compared to -0.0020 under conventional glycemic control), suggesting that intensive glycemic control may be more effective than conventional control in reducing systemic inflammation. This finding further emphasizes the importance of early and sustained good glycemic control in patients with type 2 diabetes.

Although the physiological mechanisms underlying the association between TITRE and cardiovascular outcomes remain unclear, some possible explanations have been proposed (19–21). Lower TITRE reflects poorer glycemic control, which may promote the deterioration of β-cell function. The loss of β-cells may lead to increased oxidative stress, activation of the renin-angiotensin system, release of inflammatory cytokines, and endothelial dysfunction, thereby accelerating atherosclerosis and increasing the risk of cardiovascular disease.

### Limitations

First, we did not measure other glycemic indicators, such as glycated hemoglobin (HbA1c), and therefore were unable to compare the effectiveness of TITRE and HbA1c in predicting cardiovascular disease risk and mortality. This requires further investigation. Additionally, the absence of HbA1c data may have resulted in a slightly lower number of diagnosed diabetes cases. However, our laboratory used the same diagnostic criteria in previous studies, and these criteria underwent careful review, so we believe they are reliable. Second, the need to observe a longer time window for fasting blood glucose target achievement resulted in a relatively short follow-up period. Finally, all participants were of Chinese ethnicity, which may limit the generalizability of our findings.

## Conclusion

In conclusion, our findings further emphasize the importance of maintaining good glycemic control in patients with type 2 diabetes. Long-term control of blood glucose at lower levels in patients with type 2 diabetes appears to significantly reduce the risk of diabetes-related cardiovascular outcomes and mortality.

## Data Availability

The raw data supporting the conclusions of this article will be made available by the authors, without undue reservation.

## Supplementary material

Supplementary Table 1 Sensitivity analysis of GEE results for traditional group and intensive group.
