# Supplementary material for: Traditional versus intensive blood glucose control: long-term target range duration and cardiovascular disease risk and all-cause mortality - a real-world cohort study
Source: Front Endocrinol (Lausanne). 2024 Dec 10;15:1449925. doi: 10.3389/fendo.2024.1449925 (PMC11666366; doi:10.3389/fendo.2024.1449925)
Supplement: Supplementary Table 1 — Sensitivity analysis of GEE results for traditional group and intensive group. [file Table1.docx]

**Supplementary Material**

**Supplementary Analysis: Relationship Between Glycemic Control and Inflammation**

To explore the relationship between glycemic control and inflammation, we conducted a sensitivity analysis using C-reactive protein (CRP) as a marker of inflammation. A Generalized Estimating Equation (GEE) was employed to assess the association between TITRE (calculated based on different glycemic target ranges) and repeated measurements of CRP. Given that CRP levels in our study were not normally distributed (see Supplementary Figure A), a log transformation was applied to the CRP values prior to analysis. The results, summarized in Supplementary Table S1, demonstrated a consistent negative association between TITRE and log-transformed CRP across all groups. Notably, in the intensive control group, maintaining blood glucose within lower target ranges for extended periods was associated with a reduced inflammatory response, suggesting a potential reduction in the risk of infection. These findings highlight the importance of achieving effective glycemic control to mitigate inflammation and associated risks.

| Table S1. Sensitivity analysis of GEE results for traditional group and intensive group. | | | |
| --- | --- | --- | --- |
| Variable | Estimate | 95% CI | *P* value |
| TITRE* | -0.0020 | (-0.0027, -0.0014) | < 0.0001 |
| TITRE** | -0.0025 | (-0.0033, -0.0017) | < 0.0001 |
| Adjusted for age, sex, education, smoking status, drinking status, physical activity, history of hypertension, antihypertensive medication, body mass index, systolic blood pressure, High-density lipoprotein cholesterol, Low-density lipoprotein cholesterol, estimated glomerular filtration rate, and antidiabetic medication. | | | |
| *TITRE as defined by the conventional blood glucose range. | | | |
| **TITRE as defined by the intensive blood glucose range. | | | |
