# Supplementary figures and images for: Traditional versus intensive blood glucose control: long-term target range duration and cardiovascular disease risk and all-cause mortality - a real-world cohort study
Source: Front Endocrinol (Lausanne). 2024 Dec 10;15:1449925. doi: 10.3389/fendo.2024.1449925 (PMC11666366; doi:10.3389/fendo.2024.1449925)

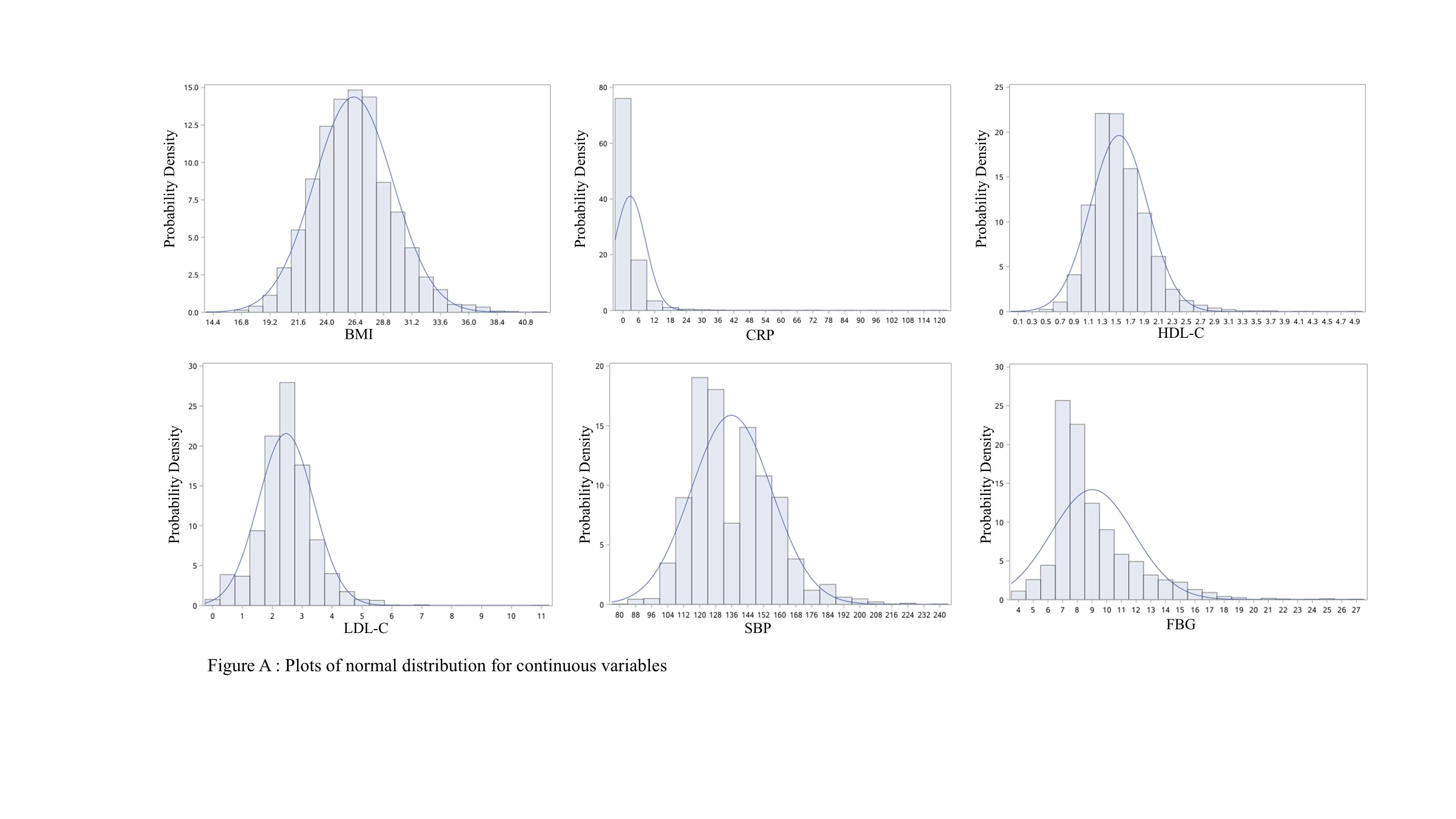

Supplement: Supplementary file 2 [file Image1.tif]
